# Supplementary material for: Prefoldin complex promotes interferon-stimulated gene expression and is inhibited by rotavirus VP3
Source: Nat Commun. 2025 Aug 29;16:8083. doi: 10.1038/s41467-025-63393-3 (PMC12397275; doi:10.1038/s41467-025-63393-3)
Supplement: Supplementary file 1 — Supplementary information [file 41467_2025_63393_MOESM1_ESM.pdf]

Prefoldin complex promotes interferon-stimulated gene expression and is inhibited by rotavirus VP3

Yinxing Zhu, Siyuan Ding et al.

Supplementary Figures and Legends

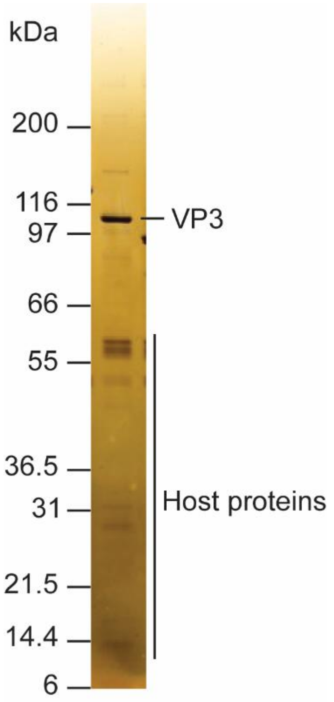

Supplementary Figure 1. Identification of host factors that interact with VP3

Silver-stained SDS-PAGE of elutes (post TEV cleavage) from an HEK293 cell line stably expressing LAP-tagged VP3.

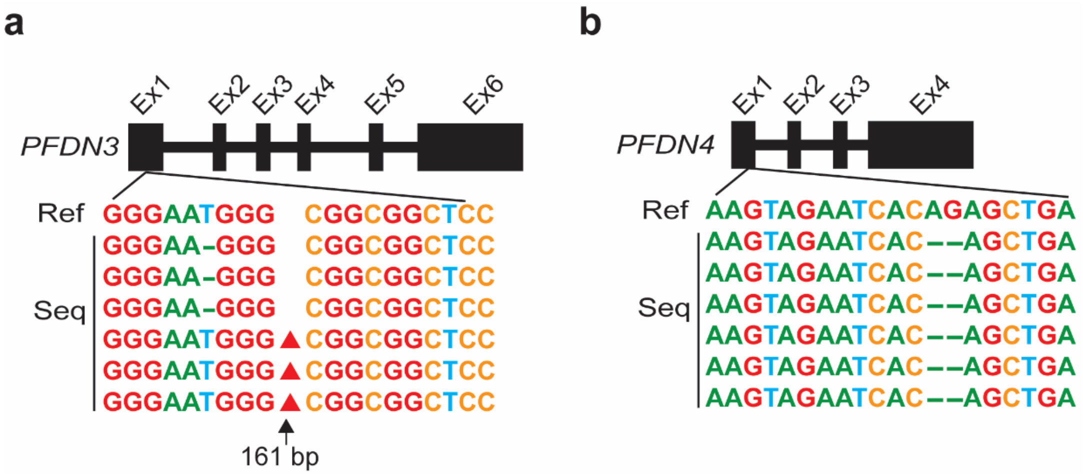

Supplementary Figure 2. *PFDN3* and *PFDN4* knockout verification

Regions of *PFDN3* (a) and *PFDN4* (b) genes targeted by sgRNAs were amplified and examined by Sanger sequencing.

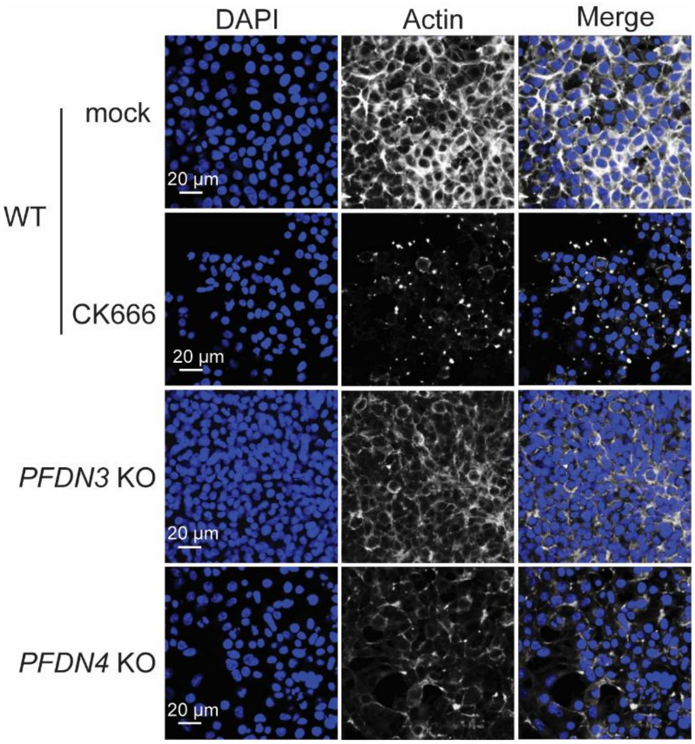

Supplementary Figure 3. Actin filament inhibition by *PFDN* knockout

Immunofluorescence analysis of actin in WT and *PFDN* KO or CK666 (100 µM) treated HEK293 cells: nucleus (DAPI, blue) and actin (actin, white). Scale bar: 20 µm.

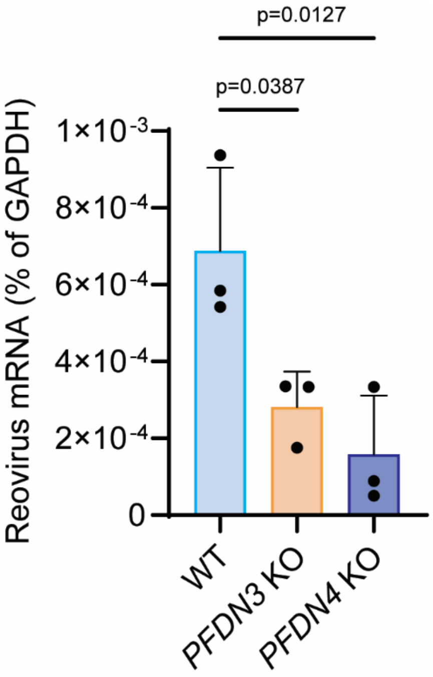

Supplementary Figure 4. Reduced reovirus replication in *PFDN* KO cells

WT, *PFDN3* KO, and *PFDN4* KO HEK293 cells were infected with reovirus (T1L strain, MOI=0.01) for 48h. Total RNA was subjected to RT-qPCR analysis. Data represents the average of three experiments; error bars indicate SEM (one-way ANOVA with Dunnett's multiple comparisons test).

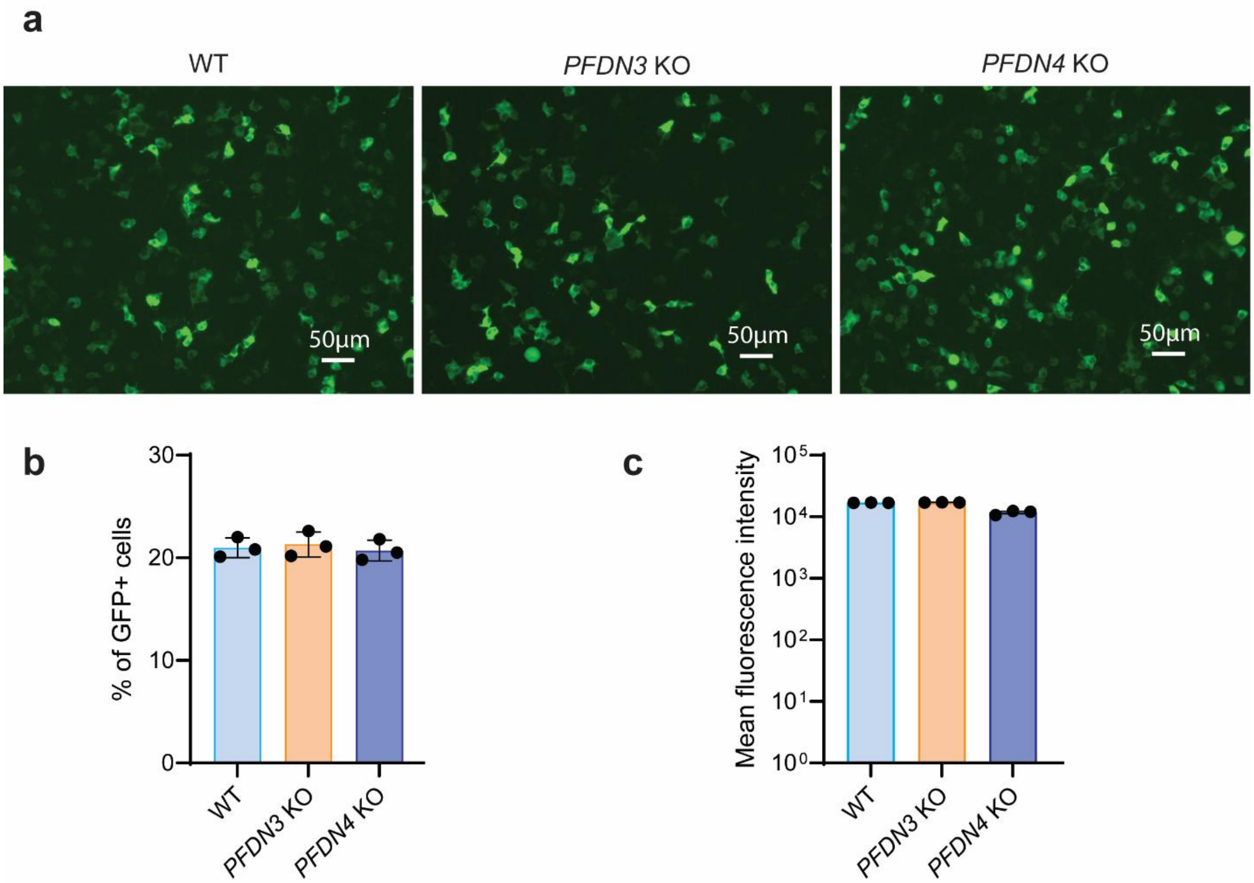

Supplementary Figure 5. Stable VP3 protein levels in *PFDN3* and *PFDN4* KO cells

WT, *PFDN3* KO, and *PFDN4* KO HEK293 cells were transfected with LAP6-VP3 plasmid for 48 hours. VP3 expression was analyzed by immunofluorescent microscopy (a) and flow cytometry (b and c).

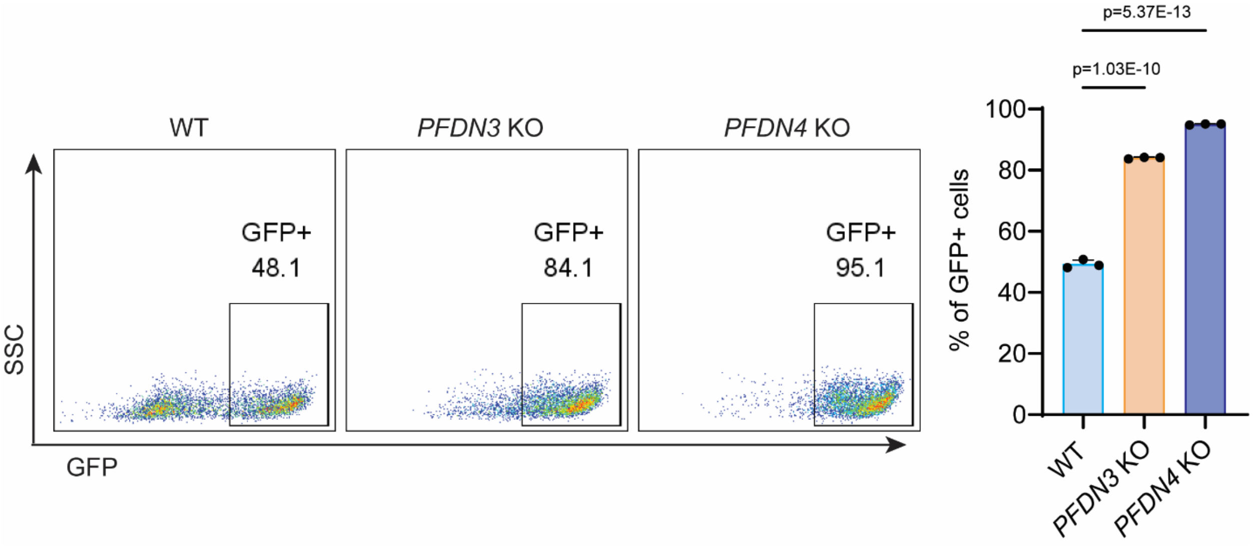

Supplementary Figure 6. Enhanced VSV-GFP virus replication in *PFDN* KO cells

WT, *PFDN3* KO, and *PFDN4* KO HEK293 cells were infected with VSV-GFP (MOI=0.01) for 24h. Percentage of GFP positive cells was quantified by flow cytometry analysis. Data represents the average of three experiments; error bars indicate SEM (one-way ANOVA with Dunnett's multiple comparisons test).

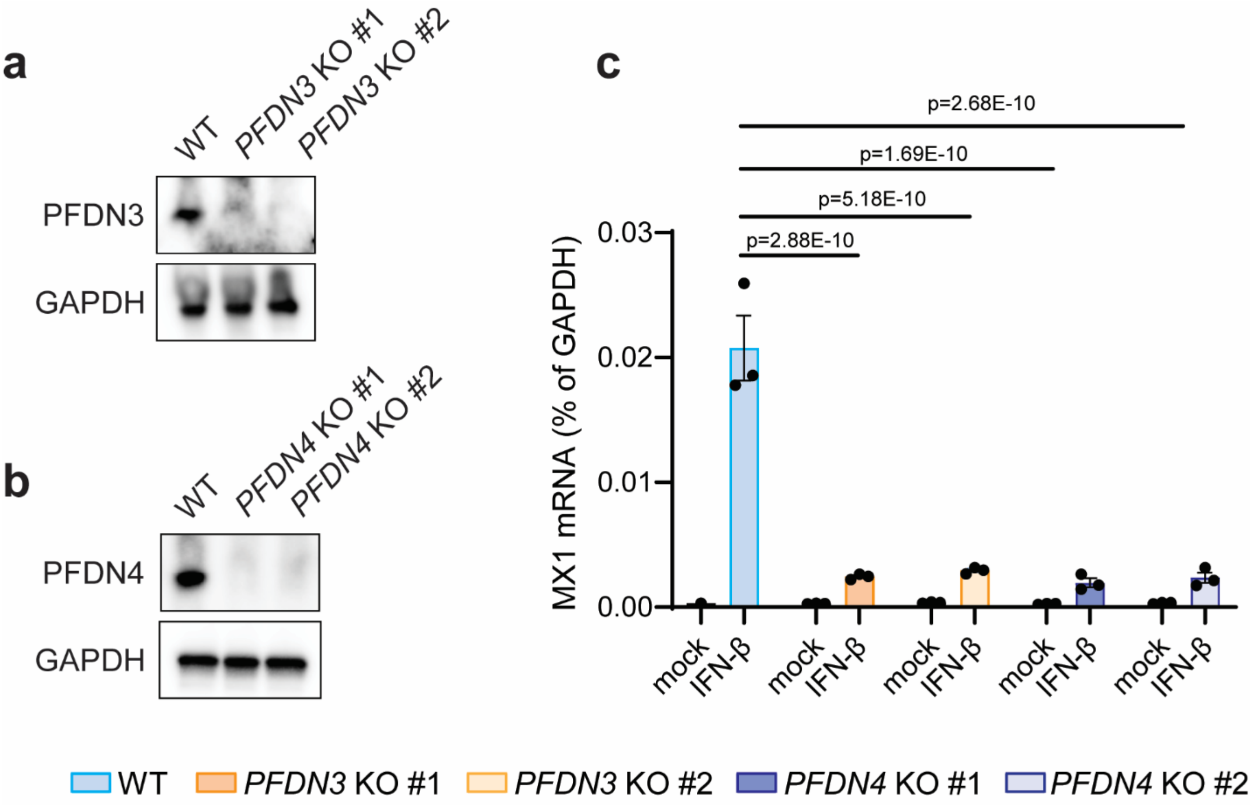

Supplementary Figure 7. Reduced ISG expression in different clones of *PFDN3* and *PFDN4* KO cells

(a) WT and *PFDN3* (clones #1, #2) HEK293 cells lysates were subjected to western blot analysis. (b) WT and *PFDN4* (clones #1, #2) HEK293 cells lysates were subjected to western blot analysis. (c) WT, *PFDN3* KO (clones #1, #2), and *PFDN4* KO (clones #1, #2) HEK293 cells were stimulated with IFN-β (500 U/ml) for 24 hours. MX1 mRNA level was measured by RT-qPCR. Data represents the average of three experiments; error bars indicate SEM (two-way ANOVA with Dunnett's multiple comparisons test).

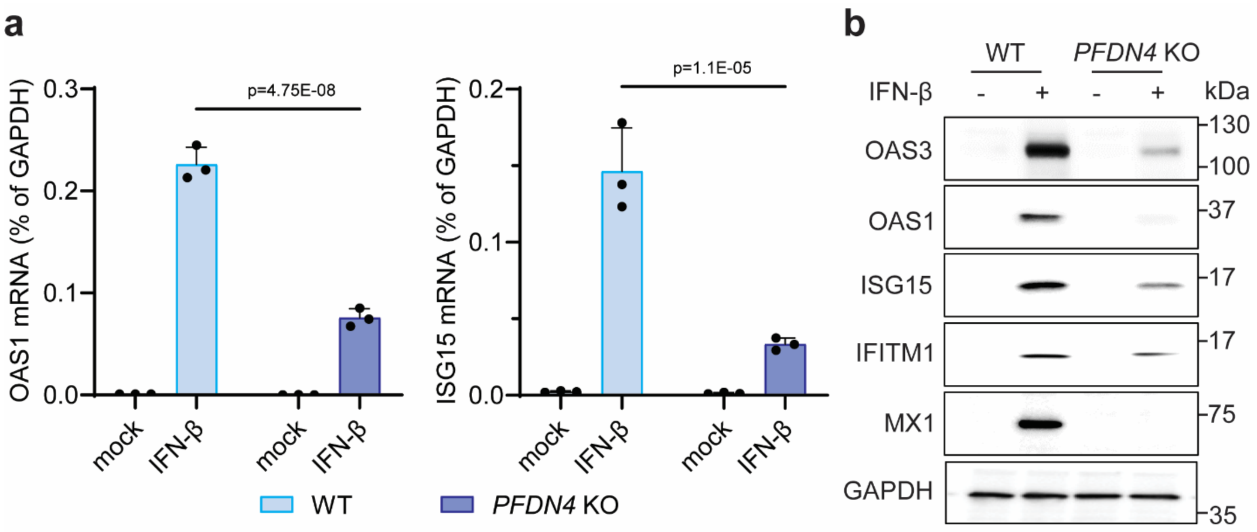

Supplementary Figure 8. Inhibited ISG production in *PFDN* KO cells

(a) WT, *PFDN3* KO, and *PFDN4* KO cells were stimulated with IFN-β (500 U/ml) for 24 hours, and OAS1 and ISG15 mRNA levels were measured by RT-qPCR. Data represents the average of three experiments; error bars indicate SEM (two-way ANOVA with Šídák's multiple comparisons test).

(b) WT, *PFDN3* KO, and *PFDN4* KO cells were stimulated with IFN-β (500 U/ml) for 24 hours, and OAS3, OAS1, ISG15, IFITM1, and MX1 protein levels were measured by western blot.

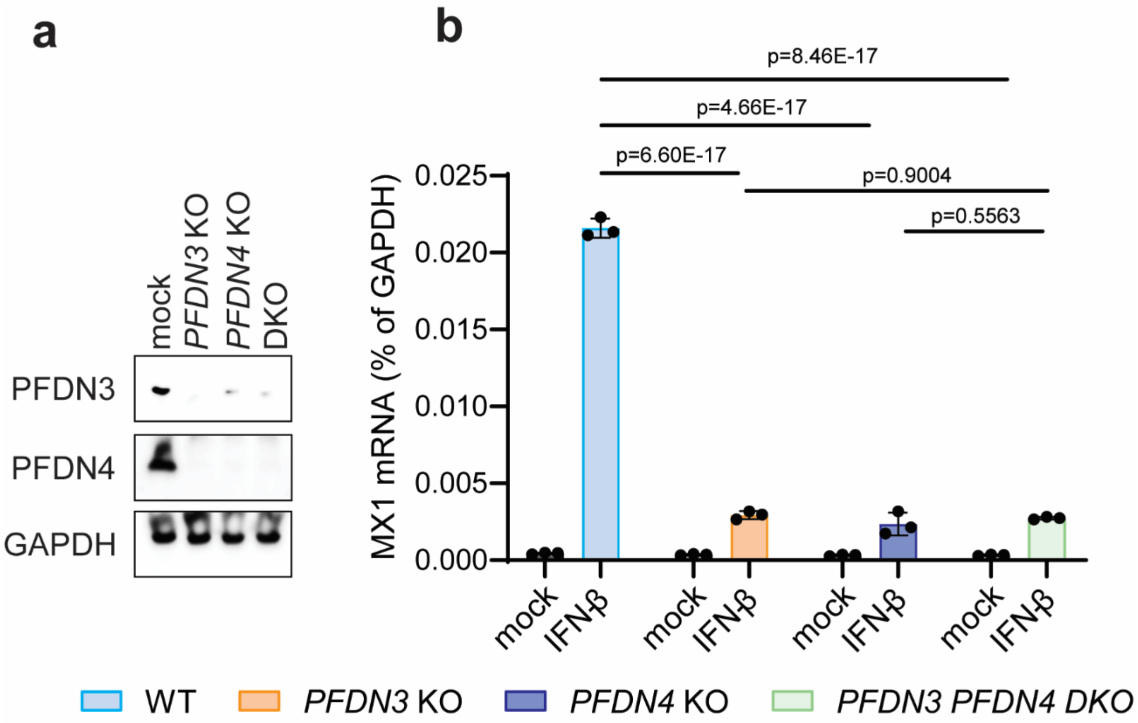

Supplementary Figure 9. Reduced ISG expression in *PFDN3* and *PFDN4* double KO cells

(a) WT, *PFDN3* KO, *PFDN4* KO, and *PFDN3* and *PFDN4* double KO cells were stimulated with IFN-β (500 U/ml) for 24 hours, and cell lysates were subjected to western blot analysis. (b) MX1 mRNA level was measured by RT-qPCR. Data represents the average of three experiments; error bars indicate SEM (two-way ANOVA with Dunnett's multiple comparisons test).

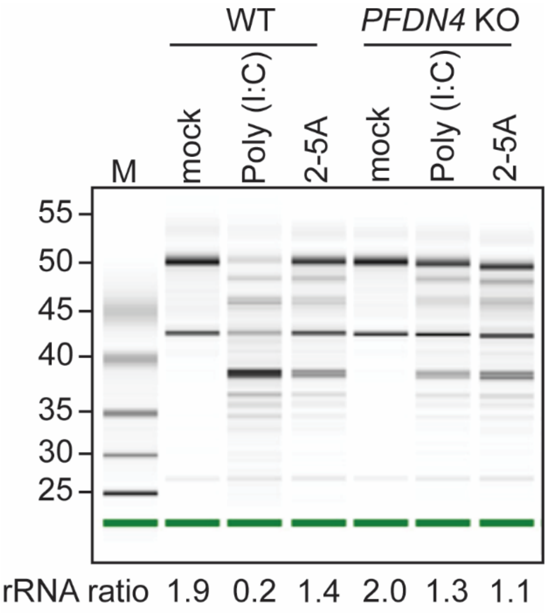

Supplementary Figure 10. Reduced rRNA degradation in *PFDN4* KO cells

WT and *PFDN4* KO cells were transfected with 4 µg/ml poly(I:C) or 2-5A for 9 hours, and total RNA was extracted and resolved by an RNA chip assay. The positions of 18S and 28S rRNAs are indicated.

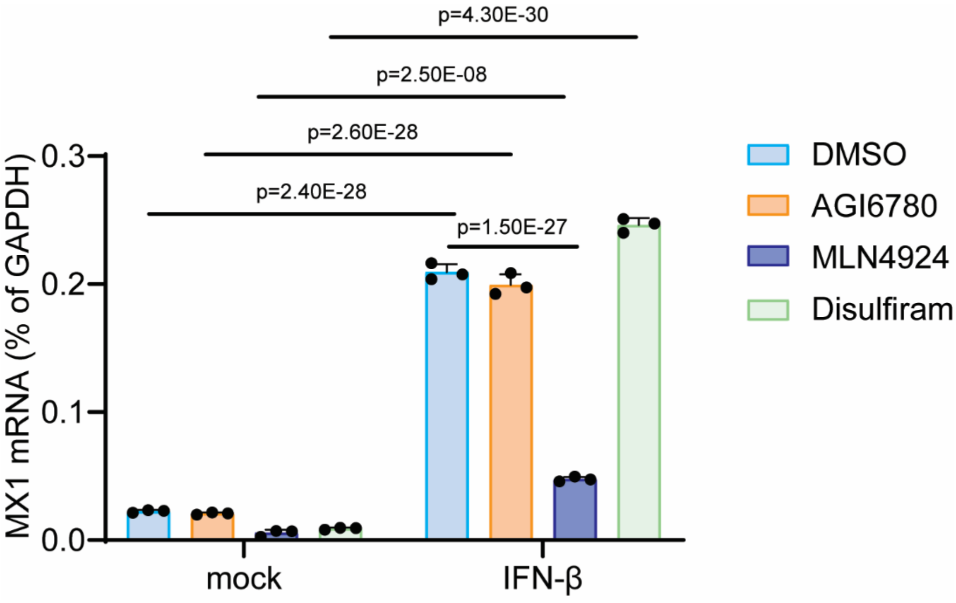

Supplementary Figure 11. Inhibited ISG expression by MLN4924 treatment

HEK293 cells were treated with DMSO, AGI6780 (10 μg/ml), MLN4924 (10 μM), or Disulfiram (10 μg/ml) for 12 hours, and then stimulated with or without IFN-β (500 U/ml) for 24 hours. MX1 mRNA was analyzed by RT-qPCR. Data represents the average of three experiments; error bars indicate SEM (two-way ANOVA with Tukey's multiple comparisons test).

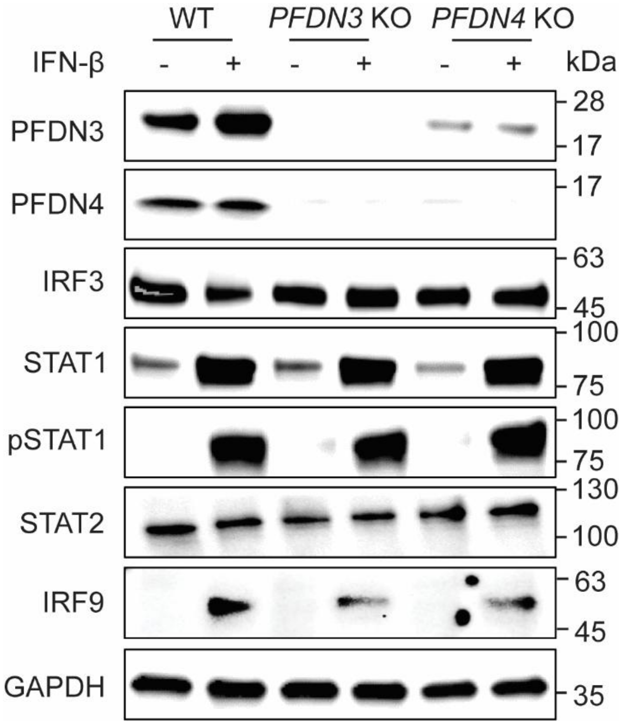

Supplementary Figure 12. Reduced IRF9 protein level in *PFDN* KO cells

WT, *PFDN3* KO, and *PFDN4* KO cells were stimulated with IFN-β (500 U/ml) for 24 hours, and cell lysates were measured by western blot.

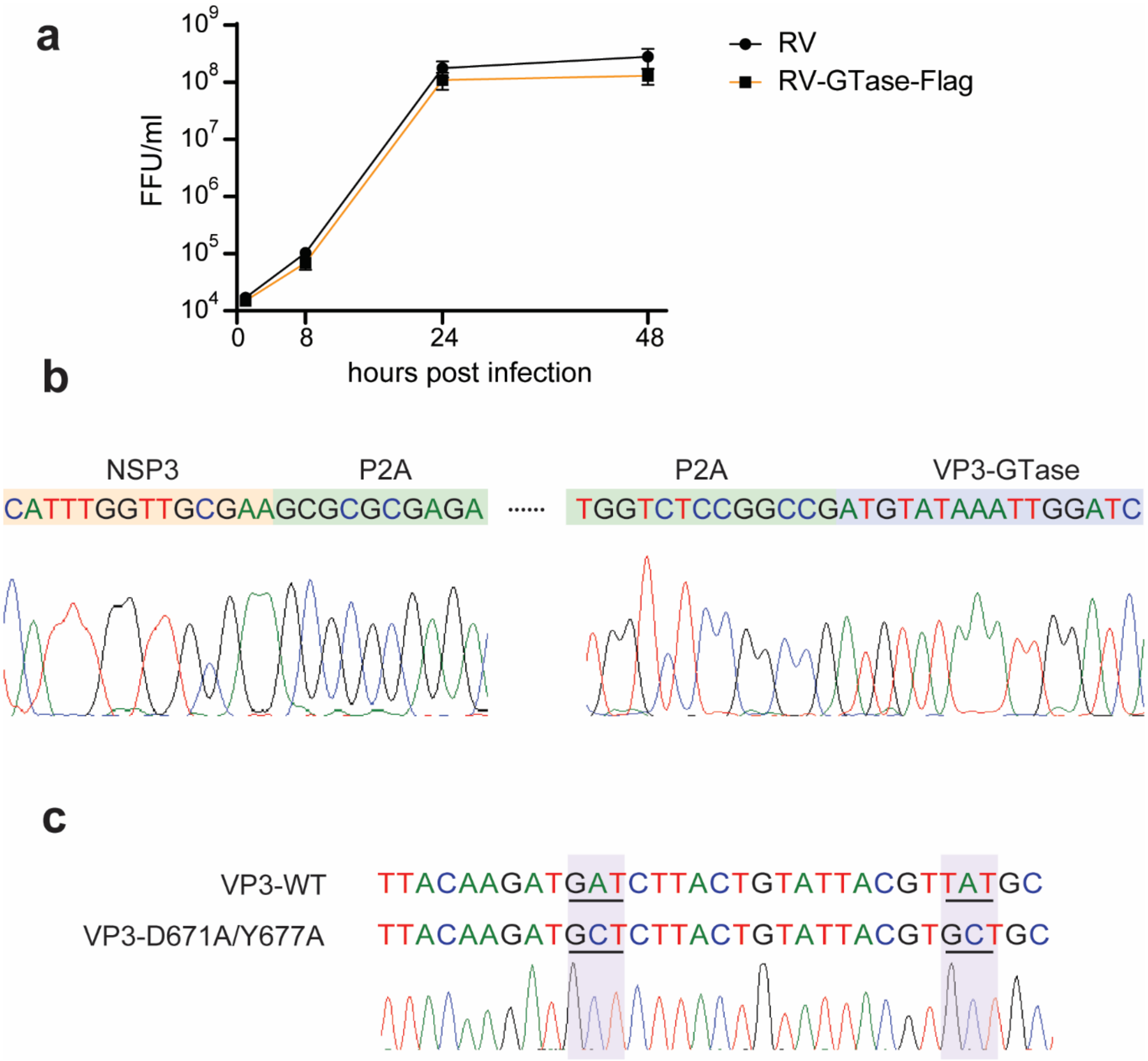

Supplementary Figure 13. Characterization of recombinant SA11 viruses with modified VP3

(a) MA104 cells were infected with SA11 and rSA11-GTase-Flag virus at an MOI of 0.01 for 1, 8, 24, and 48 h. Virus titers were determined by an FFU assay.

(b and c) Viral RNA of RV-GTase-Flag virus (b) and D671A/Y677A mutant virus (c) was extracted, and the gene segments of NSP3-P2A-VP3-GTase-Flag and VP3 were amplified for Sanger sequencing, respectively. The purple highlighted areas in (c) represent the introduced mutations.

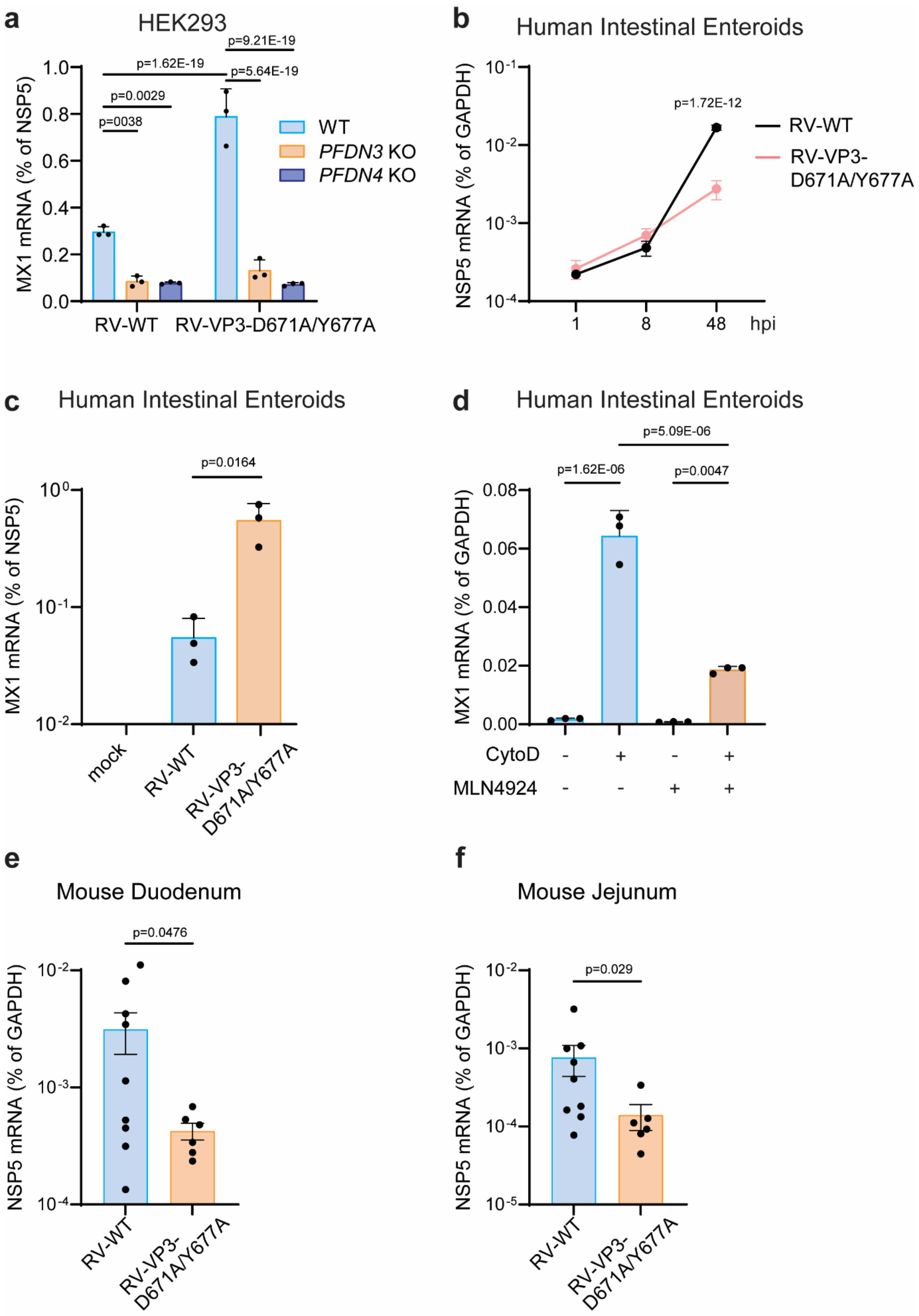
a
HEK293
MX1 mRNA (% of NSP5)
p=9.21E-19
p=1.62E-19
p=5.64E-19
p=0.0029
p=0038
WT
PFDN3 KO
PFDN4 KO
RV-WT
RV-VP3-D671A/Y677A
b
Human Intestinal Enteroids
NSP5 mRNA (% of GAPDH)
p=1.72E-12
RV-WT
RV-VP3-D671A/Y677A
hpi
c
Human Intestinal Enteroids
MX1 mRNA (% of NSP5)
p=0.0164
mock
RV-WT
RV-VP3-D671A/Y677A
d
Human Intestinal Enteroids
MX1 mRNA (% of GAPDH)
p=5.09E-06
p=1.62E-06
p=0.0047
CytoD
MLN4924
e
Mouse Duodenum
NSP5 mRNA (% of GAPDH)
p=0.0476
RV-WT
RV-VP3-D671A/Y677A
f
Mouse Jejunum
NSP5 mRNA (% of GAPDH)
p=0.029
RV-WT
RV-VP3-D671A/Y677A

Supplementary Figure 14. D671 and Y677 mutations attenuate RV infection of human intestinal epithelial cell cultures

(a) WT, PFDN3 KO, and PFDN4 KO HEK293 cells were infected with recombinant RVs expressing WT or D671A/Y677A VP3 (MOI=0.01) for 48 hours. Total RNA was detected by RT-qPCR. Data represents the average of three experiments; error bars indicate SEM (two-way ANOVA with Tukey's multiple comparisons test).

(b) Human small intestinal enteroids were infected with recombinant RVs expressing WT or RV-D671A/Y677A VP3 (MOI=0.1) for 1, 8, and 48 hours. Viral RNA was measured by RT-qPCR. Data represent the average of three experiments; error bars indicate SEM (two-way ANOVA with Šídák's multiple comparisons test).

(c) Human small intestinal enteroids were infected with recombinant RVs expressing WT or D671A/Y677A VP3 (MOI=0.1) for 1 and 48 hours. Viral RNA and MX1 RNA were measured by RT-qPCR. Data represents the average of three experiments; error bars indicate SEM (Unpaired t test).

(d) Human small intestinal enteroids were treated with CytoD (10 μg/ml), MLN4924 (1 μM), or both for 24 hours. MX1 RNA was measured by RT-qPCR. Data represents the average of three experiments; error bars indicate SEM (two-way ANOVA with Tukey's multiple comparisons test).

(e and f) Five-day-old C57BL/6 pups were infected with either WT virus (n=9) or VP3-D671A/Y677A mutant virus (n=6) for 24 hours. Small intestinal tissues, i.e., duodenum (e) and jejunum (f) were collected, and viral mRNA levels were quantified by RT-qPCR. Data represents the mean of three independent experiments; error bars indicate SEM. Statistical significance was determined by Student's t-test.

All the experiments were repeated at least three times.

Primer Name	Sequence (5′-3′)
Reovirus-F	GCATCCATTGTAAATGACGAGTCTG
Reovirus-R	CTTGAGATTAGCTCTAGCATCTTCTG
IFITM1-F	CCAAGGTCCACCGTGATTAAC
IFITM1-R	ACCAGTTCAAGAAGAGGGTGTT
MX1-F	GTGGCTGAGAACAACCTGTG
MX1-R	GGCATCTGGTCACGATCCC
OAS3-F	GCTTCAAGAGCTATGTGGACC
OAS3-R	GGAAACGTGAGTCTCAGACC
OAS1-F	TGTCCAAGGTGGTAAAGGGTG
OAS1-R	CCGGCGATTTAACTGATCCTG
ISG15-F	TCAGCCGTACCTCGAAGGTG
ISG15-R	TGGACAAATGCGACGAACCTC
GAPDH-F	GGAGCGAGATCCCTCCAAAAT
GAPDH-R	GGCTGTTGTCATACTTCTCATGG
IRF9-F	CCACCGAAGTTCCAGGTAACAC
IRF9-R	AGTCTGCTCCAGCAAGTATCGG
NSP5-F	CTGCTTCAAACGATCCACTCAC
NSP5-R	TGAATCCATAGACACGCC
NSP5-probe	CY5/TCAAATGCAGTTAAGACAAATGCAGACGCT/IABRQSP
PFDN3-seq-F	ACCACTACTGGAATGCACAGTT
PFDN3-seq-R	CAACGTGCAGTGCAGAGTGC
PFDN4-seq-F	CGAGGACAAGGAACTTGATCCCAG
PFDN4-seq-R	CCTGCCTCAGGCTCCCAAAGTGC
**sgRNA**	**Sequence (5′-3′)**
PFDN3	TGGCCACAGGGAATGGGCGG
PFDN4	AAGTAGAATCACAGAGCTGA
UBA3 #1	CTGGAGACTGGGAAGGTCGC
UBA3 #2	AAGAAGTTCCTCGAGCGATC

Supplementary Table 1. Primers and sgRNAs.
