# Supplementary material for: Prefoldin complex promotes interferon-stimulated gene expression and is inhibited by rotavirus VP3
Source: Nat Commun. 2025 Aug 29;16:8083. doi: 10.1038/s41467-025-63393-3 (PMC12397275; doi:10.1038/s41467-025-63393-3)
Supplement: Supplementary file 2 — Description Of Additional Supplementary File [file 41467_2025_63393_MOESM2_ESM.pdf]

Description of Additional Supplementary Files

File Name: Supplementary Data 1

Description: Raw data of IP-MS experiment.

File Name: Supplementary Data 2

Description: Raw data of label-free quantification (LFQ) mass spectrometry

File Name: Supplementary Data 3

Description: Raw data of RNA-seq
