# Supplementary material for: Prefoldin complex promotes interferon-stimulated gene expression and is inhibited by rotavirus VP3
Source: Nat Commun. 2025 Aug 29;16:8083. doi: 10.1038/s41467-025-63393-3 (PMC12397275; doi:10.1038/s41467-025-63393-3)
Supplement: Supplementary file 6 — Reporting summary [file 41467_2025_63393_MOESM6_ESM.pdf]

nature portfolio

Corresponding author(s): Siyuan Ding

Last updated by author(s): Aug 8, 2025

Reporting Summary

Nature Portfolio wishes to improve the reproducibility of the work that we publish. This form provides structure for consistency and transparency in reporting. For further information on Nature Portfolio policies, see our Editorial Policies and the Editorial Policy Checklist.

Statistics

For all statistical analyses, confirm that the following items are present in the figure legend, table legend, main text, or Methods section.

n/a	Confirmed	
☐	☒	The exact sample size (*n*) for each experimental group/condition, given as a discrete number and unit of measurement
☐	☒	A statement on whether measurements were taken from distinct samples or whether the same sample was measured repeatedly
☐	☒	The statistical test(s) used AND whether they are one- or two-sided<br>*Only common tests should be described solely by name; describe more complex techniques in the Methods section.*
☒	☐	A description of all covariates tested
☐	☒	A description of any assumptions or corrections, such as tests of normality and adjustment for multiple comparisons
☐	☒	A full description of the statistical parameters including central tendency (e.g. means) or other basic estimates (e.g. regression coefficient) AND variation (e.g. standard deviation) or associated estimates of uncertainty (e.g. confidence intervals)
☐	☒	For null hypothesis testing, the test statistic (e.g. *F*, *t*, *r*) with confidence intervals, effect sizes, degrees of freedom and *P* value noted<br>*Give P values as exact values whenever suitable.*
☒	☐	For Bayesian analysis, information on the choice of priors and Markov chain Monte Carlo settings
☒	☐	For hierarchical and complex designs, identification of the appropriate level for tests and full reporting of outcomes
☒	☐	Estimates of effect sizes (e.g. Cohen's *d*, Pearson's *r*), indicating how they were calculated

Our web collection on statistics for biologists contains articles on many of the points above.

Software and code

Policy information about availability of computer code

Data collection	QuantStudio Real-Time PCR Systems (ThermoFisher Scientific), LTQ-Orbitrap Elite or Fusion mass spectrometer (ThermoFisher Scientific), Preview (Protein Metrics, San Carlos), Octet-Red96 instrument (ForteBio), Illumina NovaSeq-6000, Vanquish Neo UHPLC System coupled to an Orbitrap Eclipse Tribrid Mass Spectrometer (ThermoFisher Scientific).
Data analysis	Cytoscape v3.9.1, GraphPadPrism 10, Data Analysis 9.0 software package (ForteBio), R/Bioconductor package WGCNA, FlowJo V10, MaxQuant, PyMol 2.3, Design & Analysis Software 2.8.0.

For manuscripts utilizing custom algorithms or software that are central to the research but not yet described in published literature, software must be made available to editors and reviewers. We strongly encourage code deposition in a community repository (e.g. GitHub). See the Nature Portfolio guidelines for submitting code & software for further information.

Data

Policy information about availability of data

All manuscripts must include a data availability statement. This statement should provide the following information, where applicable:

- Accession codes, unique identifiers, or web links for publicly available datasets
- A description of any restrictions on data availability
- For clinical datasets or third party data, please ensure that the statement adheres to our policy

RNA-seq raw data, please visit the Sequence Read Archive (SRA), accession number: PRJNA1164811. LFQ raw data are available via ProteomeXchange with the

identifier PXD056198. All data needed to evaluate the conclusions in the paper are present in the paper and/or the Supplementary Materials. Source data are provided with this paper.

Research involving human participants, their data, or biological material

Policy information about studies with human participants or human data. See also policy information about sex, gender (identity/presentation), and sexual orientation and race, ethnicity and racism.

Reporting on sex and gender	The authors are not aware of sex and gender of the H549 organoid.
Reporting on race, ethnicity, or other socially relevant groupings	Subjects were approached by an IRB-trained research coordinator who asked if they had a few minutes to hear about a research study that they were eligible to participate in that day. Subjects were told that tissue samples would be stored in a Biobank and could be used to generate organoid lines and for genomic research. Subjects were informed that clinical information would be collected but stored in a HIPPA compliant way and that samples will always be deidentified with a unique study ID. Subjects were informed that participation in research is completely voluntary and that they could withdraw at any time. Subjects were given a chance to voice questions or concerns. There are no potential biases present.
Population characteristics	H549 is an ileal organoid donated from a 3-month-old child with a ileostomy closure at Boston Children's Hospital Division of Endocrinology in 2019.
Recruitment	Subjects were approached by an IRB-trained research coordinator who asked if they had a few minutes to hear about a research study that they were eligible to participate in that day. Subjects were told that tissue samples would be stored in a Biobank and could be used to generate organoid lines and for genomic research. Subjects were informed that clinical information would be collected but stored in a HIPPA compliant way and that samples will always be deidentified with a unique study ID. Subjects were informed that participation in research is completely voluntary and that they could withdraw at any time. Subjects were given a chance to voice questions or concerns. There are no potential biases present.
Ethics oversight	Boston Children's Hospital IRB (P00000529)<br>Massachusetts General Hospital IRB (2003P001289)

Note that full information on the approval of the study protocol must also be provided in the manuscript.

Field-specific reporting

Please select the one below that is the best fit for your research. If you are not sure, read the appropriate sections before making your selection.

☒ Life sciences ☐ Behavioural & social sciences ☐ Ecological, evolutionary & environmental sciences

For a reference copy of the document with all sections, see nature.com/documents/nr-reporting-summary-flat.pdf

Life sciences study design

All studies must disclose on these points even when the disclosure is negative.

Sample size	The number of data points collected from each sample was based on the minimum required to perform statistical comparisons (n#3). All experiments were performed at least two independent times.
Data exclusions	No data were excluded from the analyses.
Replication	All experiments were carried out independently following the descriptions provided in the figure legends or were biologically replicated a minimum of 2-3 times. The p-values were calculated based on measurements obtained from experiments conducted independently at least three times. Representative images or results with similar numerical values were selected, and these were replicated in at least two independent biological experiments.
Randomization	The experiments were not randomized.
Blinding	The Investigators were not blinded to allocation during experiments and outcome assessment

Reporting for specific materials, systems and methods

We require information from authors about some types of materials, experimental systems and methods used in many studies. Here, indicate whether each material, system or method listed is relevant to your study. If you are not sure if a list item applies to your research, read the appropriate section before selecting a response.

Materials & experimental systems

n/a	Involved in the study
☐	☒ Antibodies
☐	☒ Eukaryotic cell lines
☒	☐ Palaeontology and archaeology
☐	☒ Animals and other organisms
☒	☐ Clinical data
☒	☐ Dual use research of concern
☒	☐ Plants

Methods

n/a	Involved in the study
☒	☐ ChIP-seq
☐	☒ Flow cytometry
☒	☐ MRI-based neuroimaging

Antibodies

Antibodies used

Antibodies used in the study included the following: rabbit anti-PFDN3 (A305-403A, Bethyl Laboratories, USA); rabbit anti-PFDN4 (16045-1-AP, Proteintech, China); mouse anti–Flag (F1804, Sigma-Aldrich, USA); mouse anti–Myc (2276S, Cell Signaling Technology, USA); rabbit anti–GAPDH (2118S, Cell Signaling Technology, USA); mouse anti–β actin (4970S, Cell Signaling Technology, USA); rabbit anti–OAS3 (PA5-59539, Invitrogen, USA); rabbit anti–OAS1 (14498S, Cell Signaling Technology, USA); rabbit anti–ISG15 (15981-1-AP, Proteintech, China); rabbit anti–IFITM1 (131263, Cell Signaling Technology, USA); rabbit anti–MX1 (13750-1-AP, Proteintech, China); rabbit anti–IRF3 (4302S, Cell Signaling Technology, USA); rabbit anti–STAT1 (9172S, Cell Signaling Technology, USA); rabbit anti–pSTAT1 (9174S, Cell Signaling Technology, USA); rabbit anti-STAT2 (sc-514193, Santa Cruz Biotechnology, USA); mouse anti–UBA3 (sc-377272, Santa Cruz Biotechnology, USA); rabbit anti–IRF9 (76684, Cell Signaling Technology, USA); horseradish peroxidase–conjugated anti-mouse and anti–rabbit (7076S and 7074S, cell signaling technology, USA); Alexa Fluor 594–conjugated goat anti-mouse (A-11005, Thermo Fisher Scientific, USA); 4′,6′-diamidino-2-phenylindole (1:1000) (00-4959-52, Invitrogen, USA).

Validation

Validation is available on the manufacturer´s website.

Eukaryotic cell lines

Policy information about cell lines and Sex and Gender in Research

Cell line source(s)

HEK293T (ATCC, CRL-11268); MA104 cells (CRL-2378, ATCC); HT-29 (HTB-38, ATCC); A549 (CRM-CCL-185, ATCC); BHK-T7 cell line was provided by Dr. Ursula Buchholz (Laboratory of Infectious Diseases, NIAID, NIH, USA), SERPINB1 knockout cells were generated in our lab (Yinxing Zhu et al. npj Vaccines, 2024). PFDN3 knockout, PFDN4 knockout, PFDN3 and PFDN4 knockout rescue, UBA3 knockout, and UBA3 and PFDN4 double knockout HEK293 cells were confirmed by western blot.

Authentication

Cultures were tested for Mycoplasma contamination using the e-Myco plus mycoplasma PCR detection kit following the manufacturer's manual (Boca Scientific).

Mycoplasma contamination

Cultures were tested for Mycoplasma contamination using the e-Myco plus mycoplasma PCR detection kit following the manufacturer's manual (Boca Scientific).

Commonly misidentified lines
(See ICLAC register)

no

Animals and other research organisms

Policy information about studies involving animals; ARRIVE guidelines recommended for reporting animal research, and Sex and Gender in Research

Laboratory animals

C57BL/6 mice were purchased from the Jackson Laboratory for breeding.

Wild animals

Provide details on animals observed in or captured in the field; report species and age where possible. Describe how animals were caught and transported and what happened to captive animals after the study (if killed, explain why and describe method; if released, say where and when) OR state that the study did not involve wild animals.

Reporting on sex

We have not observed any sex-dependent phenotypes in rotavirus susceptibility in vivo, therefore we used both male and female mice in the current study.

Field-collected samples

For laboratory work with field-collected samples, describe all relevant parameters such as housing, maintenance, temperature, photoperiod and end-of-experiment protocol OR state that the study did not involve samples collected from the field.

Ethics oversight

All animal studies were approved by Washington University in St. Louis Institutional Animal Care and Use Committee (IACUC) with the protocol number 22-0269.

Note that full information on the approval of the study protocol must also be provided in the manuscript.

Plants

Seed stocks

Report on the source of all seed stocks or other plant material used. If applicable, state the seed stock centre and catalogue number. If plant specimens were collected from the field, describe the collection location, date and sampling procedures.

Novel plant genotypes

Describe the methods by which all novel plant genotypes were produced. This includes those generated by transgenic approaches, gene editing, chemical/radiation-based mutagenesis and hybridization. For transgenic lines, describe the transformation method, the number of independent lines analyzed and the generation upon which experiments were performed. For gene-edited lines, describe the editor used, the endogenous sequence targeted for editing, the targeting guide RNA sequence (if applicable) and how the editor was applied.

Authentication

Describe any authentication procedures for each seed stock used or novel genotype generated. Describe any experiments used to assess the effect of a mutation and, where applicable, how potential secondary effects (e.g. second site T-DNA insertions, mosiacism, off-target gene editing) were examined.

Flow Cytometry

Plots

Confirm that:

☒ The axis labels state the marker and fluorochrome used (e.g. CD4-FITC).

☒ The axis scales are clearly visible. Include numbers along axes only for bottom left plot of group (a 'group' is an analysis of identical markers).

☒ All plots are contour plots with outliers or pseudocolor plots.

☒ A numerical value for number of cells or percentage (with statistics) is provided.

Methodology

Sample preparation

VSV-GFP virus infected HEK293 cells or VP3-GFP transfected HEK293 cells were collected, and single cell suspension was prepared following the protocol from the manufacturer.

Instrument

Infected cells were analyzed using a BD LSR Fortessa Flow Cytometer.

Software

Data were analyzed using FlowJo.

Cell population abundance

For VSV-GFP infected samples, >45% of collected events gated as HEK293 cells.

Gating strategy

Doublets were removed by FSC-A/FSC-H. GFP positive cells were gated through SSC-A/FITC.

☐ Tick this box to confirm that a figure exemplifying the gating strategy is provided in the Supplementary Information.
